# Context-Dependent Roles of Rnd3 in Cancer: Revisiting a Functional Paradox

**DOI:** 10.3390/cells15171602

**Published:** 2026-09-03

**Authors:** Elisa Lledó, Olga Gómez, Alexandra Bizy, Amalia Solana-Orts, José Terrado, Begoña Ballester-Lurbe, Enric Poch

**Affiliations:** 1 Department of Biomedical Sciences, School of Health Sciences, Universidad Cardenal Herrera-CEU, CEU Universities, 46115 Alfara del Patriarca, Spain; 2 Department of Animal Medicine and Surgery, Facultad de Veterinaria, Universidad Cardenal Herrera-CEU, CEU Universities, 46115 Alfara del Patriarca, Spain

**Keywords:** Rnd3/RhoE, Rho GTPases, cancer, RhoA, ROCK, epithelial–mesenchymal plasticity, apoptosis, therapy resistance

## Abstract

**Highlights:**

**What are the main findings?**

- Rnd3 exerts a conserved molecular action across tumors: inhibition of RhoA/ROCK1-dependent contractility.
- A single Rnd3-driven inhibition of RhoA/ROCK1 bifurcates into opposite tumor outcomes via distinct downstream effectors.

**What are the implications of the main findings?**

- Context-specific accessory effectors, not tumor lineage alone, redirect this conserved action.
- Predicting whether Rnd3 acts as a suppressor or facilitator requires measuring the pre-existing cellular and mechanical state, rather than relying on tumor lineage or Rnd3 levels alone.

**Abstract:**

Rnd3 is an atypical member of the Rho GTPase family whose activity is mainly regulated by expression, localization and protein stability rather than canonical GDP/GTP cycling. In cancer, Rnd3 has been described both as a tumor suppressor and as a tumor-promoting factor, creating an apparent functional paradox. We propose that this paradox is resolved by a mechanistic invariant: Rnd3 exerts a conserved inhibition of RhoA/ROCK1-dependent actomyosin contractility, whose phenotypic output is redirected by context-specific accessory effectors rather than reversed. In this review, we revisit this paradox by integrating evidence from mechanistic studies, tumor models and patient-associated datasets. We propose that Rnd3 should not be interpreted through a binary oncogene/tumor-suppressor framework, but rather as a context-dependent regulator of tumor cell state. In many tumor settings, Rnd3 repression or loss of Rnd3 function favors proliferation, apoptosis resistance and therapy resistance through pathways involving Notch, NF-κB, EGFR/ERK, EZH2-dependent chromatin regulation, m6A-mediated RNA control, microRNAs and chaperone-mediated autophagy. However, in selected contexts, including RTK-driven glioblastoma, hepatocellular carcinoma, non-small-cell lung cancer, melanoma and gastric cancer, Rnd3 may support tumor fitness, migration or invasive plasticity. We therefore propose a functional stratification model in which Rnd3 output depends on the biological process, tumor lineage, pathway activity and mechanical state of the cell.

## 1. Introduction

Members of the Rho family of small GTPases coordinate cytoskeletal organization, cell polarity, adhesion, migration, proliferation and survival, processes that are frequently altered during tumor initiation and progression [1]. Rnd3, encoded by the RND3 gene, belongs to the atypical Rnd subgroup and differs from canonical Rho GTPases because it remains predominantly GTP-bound and displays negligible intrinsic GTPase activity [2]. Consequently, Rnd3 does not operate as a conventional GDP/GTP molecular switch. Instead, its functional output is determined largely by RND3 expression and by the subcellular localization, post-translational modification and turnover of the Rnd3 protein. Farnesylation contributes to its membrane association, whereas phosphorylation by ROCK1 or PKCα regulates Rnd3 stability, localization and activity [2,3,4]. Phosphorylation-dependent binding to 14-3-3 proteins promotes redistribution of Rnd3 from the plasma membrane to the cytosol, while Skp2-mediated ubiquitylation targets Rnd3 for proteasomal degradation during cell-cycle progression [5,6].

**Figure 1:**
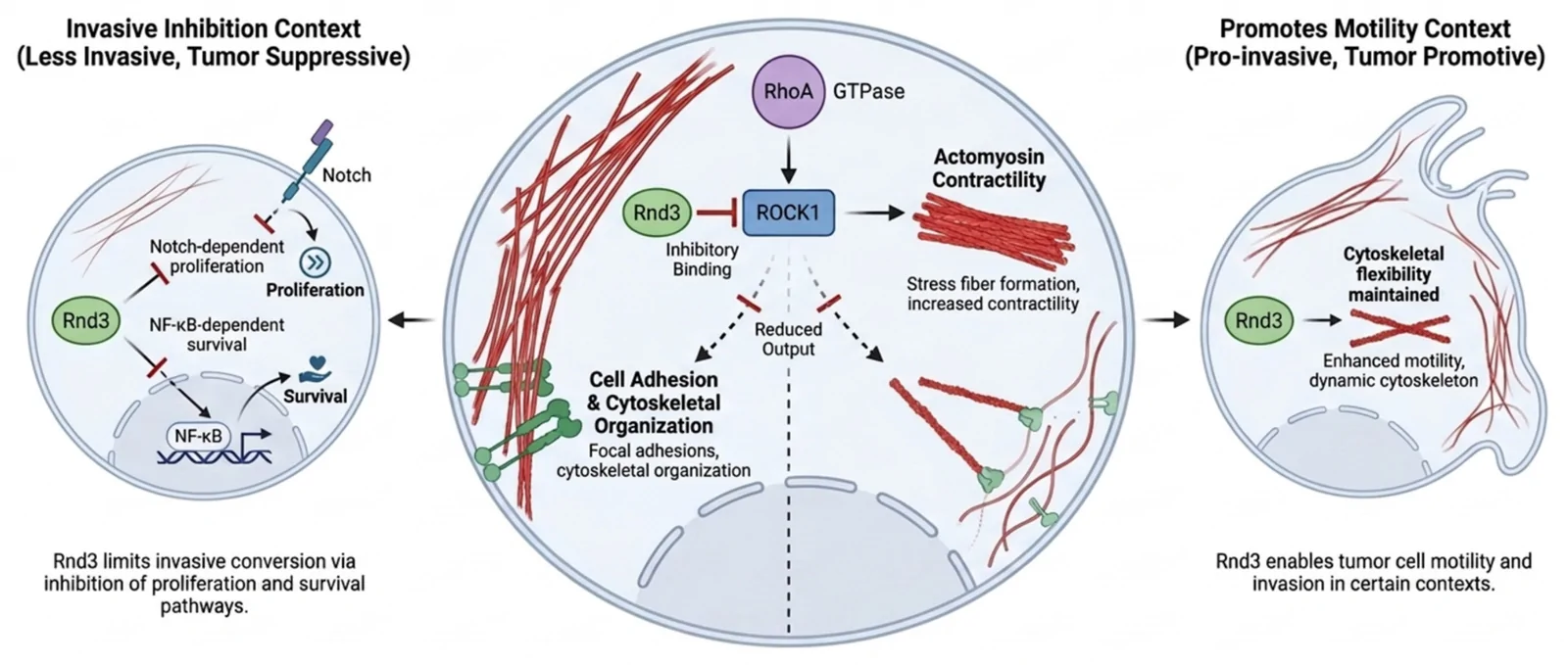
Rnd3 as a conserved inhibitor of RhoA/ROCK1-dependent actomyosin contractility with context-dependent outcomes in cancer. At the core of the mechanism, RhoA-activated ROCK1 promotes actomyosin contractility, stress-fiber formation and focal-adhesion organization; Rnd3 binds and inhibits ROCK1, reducing this canonical output. The same molecular action yields divergent results depending on the cellular context (side panels). In a tumor-suppressive context (**left**), Rnd3 limits invasive conversion by restraining Notch-dependent proliferation and NF-κB-dependent survival. In a pro-invasive context (**right**), Rnd3 preserves the cytoskeletal flexibility required for tumor cell motility and invasion. The figure illustrates the basic mechanism only; the direction of effect in each setting depends on the pre-existing cellular state and accessory effectors, as developed in the main text.

Rnd3 is best characterized as an endogenous antagonist of RhoA/ROCK-dependent actomyosin contractility. By directly binding ROCK1 and inhibiting downstream signaling, Rnd3 limits stress-fiber formation and modulates adhesion and cytoskeletal organization [7]. However, this molecular activity does not generate a single invariant phenotype in cancer. In some tumor contexts, Rnd3 inhibits Notch-dependent proliferation or NF-κB-dependent survival [8,9,10] and limits invasive conversion [11,12]. In others, Rnd3 preserves the cytoskeletal flexibility required for tumor cell motility and invasion [13]. Moreover, treatment-induced downregulation of RND3 can activate compensatory RhoA/ROCK-dependent signaling and metabolic programs that promote therapeutic resistance [14]. Thus, the functional output of Rnd3 depends not only on its expression levels, but also on the dominant signaling pathway, mechanical state and selective pressure operating in each tumor context. This distinction between an invariant molecular action and a context-dependent phenotypic output is the central argument of this review: although the inhibition of RhoA/ROCK1 signaling remains constant, context-specific accessory effectors determine whether this conserved action manifests as tumor suppression, facilitation or plasticity (Figure 1).

**Figure 2:**
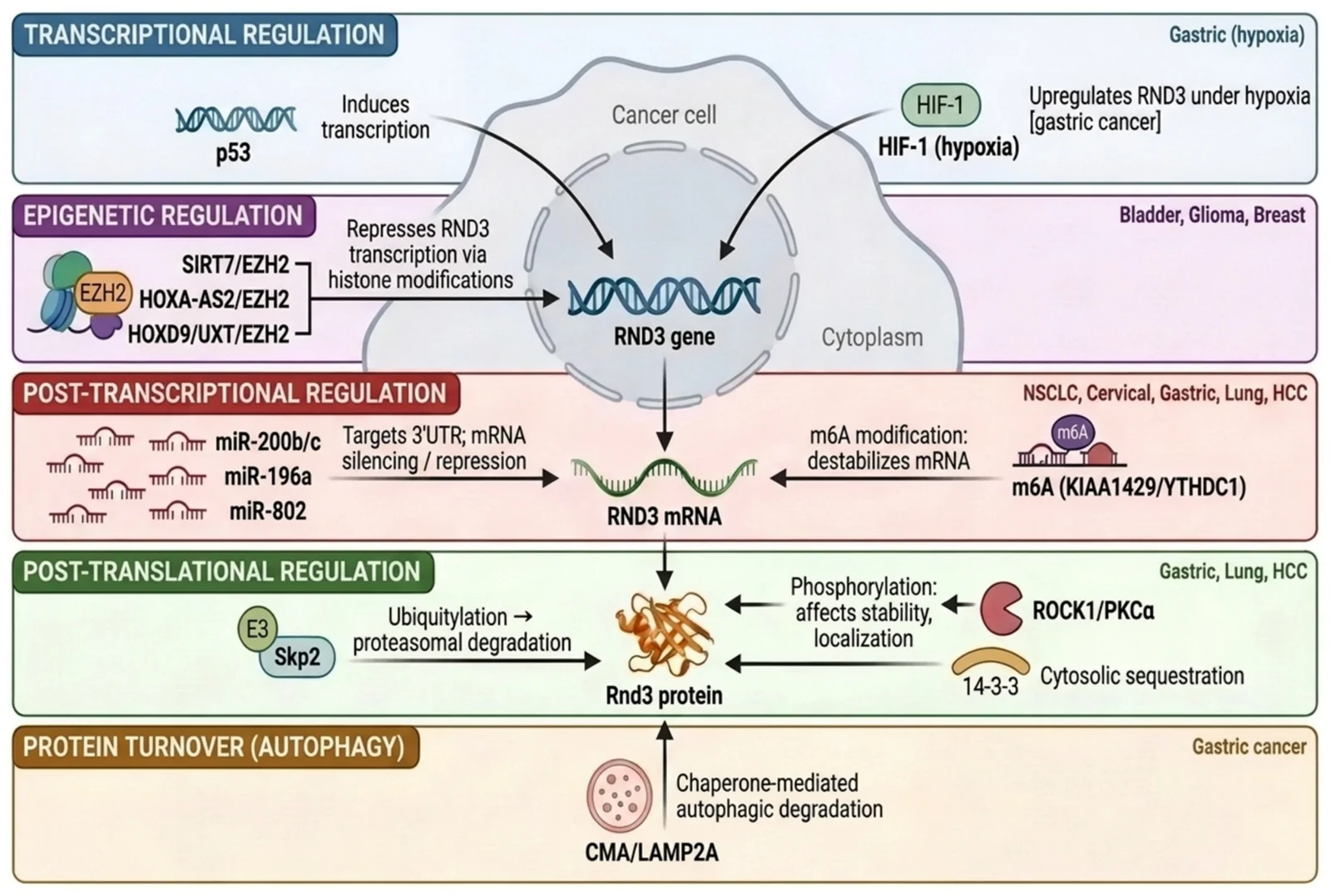
Multilayered regulation of RND3 expression and Rnd3 protein abundance in cancer. As an atypical Rho GTPase that remains predominantly GTP-bound and lacks a functional GDP/GTP cycle, Rnd3 is controlled mainly through the amount, localization and turnover of the protein. At the transcriptional level, p53 and, under hypoxia, HIF-1 induce RND3 expression, whereas EZH2-containing complexes recruited by SIRT7, HOXA-AS2 or HOXD9–UXT epigenetically repress it. Post-transcriptionally, RND3 mRNA is silenced by microRNAs (miR-200b/c, miR-196a, miR-802) and destabilized by m6A modification (KIAA1429/YTHDC1). At the protein level, Rnd3 abundance and localization are modulated by Skp2-mediated proteasomal degradation, ROCK1/PKCα phosphorylation, 14-3-3 sequestration and LAMP2A-dependent chaperone-mediated autophagy. The balance among these layers sets the level of active Rnd3, providing a mechanistic basis for its context-dependent functions. Gene symbol (RND3) denotes transcript-level events; protein symbol (Rnd3) denotes protein-level function. Abbreviations: CMA, chaperone-mediated autophagy; HIF-1, hypoxia-inducible factor 1; m6A, N6-methyladenosine; 3′UTR, three-prime untranslated region.

This context dependence is reinforced by the multiple regulatory mechanisms through which cancer cells modify RND3 expression or Rnd3 protein levels. RND3 transcription can be induced by p53 [15] and repressed through tumor-specific chromatin-regulatory mechanisms involving SIRT7/EZH2 [16], HOXA-AS2/EZH2 [17] or the HOXD9–UXT–EZH2 axis [18]. At the post-transcriptional level, RND3 mRNA is targeted by regulatory microRNAs, including miR-200b/c, and can be destabilized through KIAA1429/YTHDC1-dependent m6A modification [19,20]. Rnd3 protein levels are further controlled by Skp2-dependent proteasomal degradation and, in gastric cancer cells, by LAMP2A-dependent chaperone-mediated autophagy [6,21]. These regulatory layers alter the amount, localization and cellular availability of Rnd3. At the level of localization, membrane-associated Rnd3 sustains its canonical inhibition of ROCK1, whereas phosphorylation-dependent binding by 14-3-3 proteins displaces Rnd3 to the cytosol and attenuates this activity [5]. However, this membrane-versus-cytosol distribution modulates the intensity of the canonical action rather than determining, on its own, whether Rnd3 acts as a suppressor or a facilitator of tumor progression. Together, these mechanisms provide a mechanistic basis for its divergent functions across tumor types (Figure 2).

In 2016, Paysan et al. reviewed alterations in RND3 expression and Rnd3 function across different cancers, framing its role within the classical dichotomy of tumor suppressor versus tumor promoter [22]. That review established the dual behavior of Rnd3 and its dependence on tumor type and experimental context. Building on this concept, the present review does not aim to reclassify Rnd3 as either oncogenic or tumor-suppressive. Instead, we revisit its role according to the main biological processes in which it participates—including proliferation, apoptosis, therapeutic response, migration, invasion and epithelial–mesenchymal plasticity—and examine how multilayered regulation, signaling architecture and cellular state determine its functional output.

### Defining Biological Context for Rnd3 Function

Because “context” is central to this review, it requires an explicit, non-circular definition. We define it as the experimentally measurable molecular, cellular, microenvironmental and therapeutic state present before or during Rnd3 perturbation, characterized independently of the resulting phenotype. This independence is what distinguishes a defining condition from the phenotype it explains. So defined, context is not equivalent to tumor lineage: opposing results within one tumor type show that lineage alone is insufficient, and that measurable variables—baseline RhoA/ROCK activity, dominant signaling pathway, cellular state and prior therapeutic exposure—may better account for divergent Rnd3 outputs. This is illustrated within a single tumor, glioblastoma, where Rnd3 restrains Notch- and NF-κB-driven progression in some models [10,11] yet acts as a pro-invasive effector downstream of receptor tyrosine kinase signaling in others [13]. Consistently, in non-transformed settings Rnd3 switches between pro- and anti-apoptotic roles depending on cell lineage and physiological state [23]. Establishing that a variable genuinely constitutes context therefore requires evidence that the effect of Rnd3 changes when that variable changes: it is not enough that Rnd3 accompanies invasion under a given condition, since the direction of its effect must shift when the pre-existing state does. Within RTK-driven glioblastoma models, this criterion is met, as sustained RTK signaling maintains Rnd3 expression, and both its silencing and its overexpression causally reshape the invasive phenotype through RhoA/ROCK [13]; what remains untested is whether the same manipulation reverses Rnd3 direction across the opposing, Notch-restraining models, so that RhoA/ROCK activity, dominant pathway and therapeutic history are treated here as candidate context variables that predict, rather than merely accompany, the direction of Rnd3 function.

## 2. Rnd3 in Tumor Proliferation and Growth Control

Cell cycle control and proliferation are critical pathways that are systematically disrupted in tumor cells. In tumor studies, Rnd3 has been generally characterized by its anti-proliferative capacity, yet other studies have documented it as a tumor-promoting protein. In non-small-cell lung cancer, Rnd3 suppresses proliferation independently of ROCK by promoting proteasomal degradation of NICD, thereby inhibiting the Notch/Hes1 axis [8]. Liu et al. (2015) described a similar antiproliferative mechanism in glioblastoma, showing that Rnd3 is downregulated in this tumor and that its overexpression suppresses cell proliferation and xenograft growth by inhibiting Notch transcriptional activity [9]. Together, these studies support the idea that Rnd3 can constrain tumor growth by limiting Notch-dependent proliferative programs. In apparent contrast, network-based analyses of high-grade glioma reported that Rnd3 is up-regulated rather than lost and identified it as a key positive regulator of tumor proliferation, migration, invasion and angiogenesis [24]. This opposite directionality—Rnd3 loss being tumor-suppressive in some glioma models yet RND3 overexpression being tumor-promoting in others—indicates that the effect of Rnd3 on glioma growth is not uniform and depends on tumor grade and the dominant regulatory network. The tumor-suppressive arm of this balance is further supported by Wu et al. [17], who showed that the lncRNA HOXA-AS2 promotes glioma progression in part through repression of RND3: HOXA-AS2 knockdown reduced proliferation and colony formation in human glioma cell lines, whereas direct interference with RND3 partially rescued these effects, supporting RND3 as a functional mediator of the antiproliferative response.

In colorectal carcinoma, miR-17 promotes cell proliferation, tumor growth and cell-cycle progression by directly targeting the RND3 tumor-suppressor gene, providing functional evidence that post-transcriptional repression of RND3 can contribute to colorectal tumor growth [25].

Several additional tumor types reinforce the idea that cancer cells may increase proliferative capacity by suppressing Rnd3. In bladder cancer, SIRT7 and EZH2 cooperate to repress RND3, and reduced Rnd3 contributes to increased viability, clonogenicity and cisplatin resistance [16]. In breast cancer, the HOXD9/UXT/EZH2 axis represses RND3 and supports proliferation, invasion and tumor growth; conversely, restoration of RND3 contributes to the antitumor effects observed when this axis is inhibited [18]. In gastric cancer, Rnd3 is controlled at the protein-stability level: LAMP2A-dependent chaperone-mediated autophagy degrades Rnd3 and thereby sustains proliferative capacity. Inhibition of chaperone-mediated autophagy increases Rnd3 levels and reduces proliferation, while Rnd3 silencing partially rescues the growth arrest induced by LAMP2A depletion [21]. Together, these studies show that different regulatory mechanisms, including chromatin repression and selective protein degradation, can converge on reduced Rnd3 activity and increased tumor growth. Consistent with this suppressive role, Rnd3 overexpression also inhibits tumor cell growth and promotes apoptosis in esophageal squamous cell carcinoma, at least partly through reduced EGFR signaling and ERK phosphorylation [26]. When considered alongside the Notch-dependent mechanisms described in glioblastoma and NSCLC, these findings support the view that Rnd3 does not suppress proliferation through a single universal pathway. Rather, Rnd3 acts as a context-dependent antiproliferative node whose upstream regulation and downstream effectors vary according to tumor lineage and cellular state.

While Rnd3 has been investigated in tumor biology as mainly an anti-proliferative protein, other studies have also described its opposing oncogenic role. The main boundary conditions to the antiproliferative model come from hepatocellular carcinoma and non-small-cell lung cancer. In hepatocellular carcinoma, Rnd3 acts as a reversible modulator of cell cycle homeostasis, where its depletion drives cells into a transient, hTERT-depleted senescent state, while its restoration successfully reverses this phenotype to recover proliferative capacity [27]. In non-small-cell lung cancer, the microRNA miR-200b/c directly silences RND3 expression by targeting its 3′UTR. Consequently, miR-200b/c overexpression, which mimics Rnd3 knockdown, reduces cell proliferation and invasion, indicating that Rnd3 is required to sustain malignant behavior in these tumors [19]. This finding appears to contrast with their earlier NSCLC Notch study, where Rnd3 reintroduction inhibited proliferation [8]. Tang and colleagues justified this discrepancy by citing cell lineage limitations in their initial models, proposing a temporal model where Rnd3 is down-regulated during tumor initiation but actively up-regulated during advanced progression.

Taken together, the available evidence supports Rnd3 as a frequent, but not universal, suppressor of tumor proliferation. The strongest tumor-suppressive evidence comes from models of glioblastoma/glioma, colorectal carcinoma, bladder cancer, breast cancer, gastric cancer and esophageal squamous cell carcinoma, where Rnd3 loss, repression or degradation supports proliferation, clonogenic growth, cell-cycle progression or tumor expansion. Mechanistically, these effects involve Notch inhibition, Rb/cyclin D1/ERK regulation, EZH2-dependent chromatin repression, lncRNA-mediated regulation, EGFR/ERK attenuation, miRNA-mediated targeting and chaperone-mediated autophagic degradation. In contrast, HCC, selected glioma models and some NSCLC contexts define important exceptions in which Rnd3 may be required to maintain proliferative competence, prevent senescence or support malignant behavior. Therefore, Rnd3 should not be described as a uniformly acting proliferation suppressor. Rnd3 frequently limits tumor growth, although its proliferative output remains conditional on tumor lineage, oncogenic circuitry and cellular state. Across the studies reviewed here, tumor-suppressive outputs have been reported more frequently than pro-tumoral ones, although this likely reflects the contexts examined to date rather than an intrinsic hierarchy of Rnd3 functions.

## 3. Rnd3 in Apoptosis and Therapeutic Response

The role of Rnd3 in cell survival is closely connected to its antiproliferative activity. In several cancer models, reduced RND3 expression is associated with resistance to apoptosis, enhanced survival signaling and impaired response to anticancer therapy [28,29]. Consistently, restoration or functional maintenance of Rnd3 can promote apoptotic competence or resensitize tumor cells to treatment [10,16,30]. Hence, as observed for proliferation, the role of Rnd3 is not universal and suggests that it often limits tumor cell survival, but its final output depends on the oncogenic circuitry, the dominant death pathway and the therapeutic context.

### 3.1. Rnd3 and Apoptotic Competence

Early evidence supporting a pro-apoptotic role of Rnd3 came from prostate cancer. In 2005 Bektic et al. reported that Rnd3 is underexpressed in prostate cancer and that its re-expression induces cell-cycle arrest and apoptosis [28]. A similar survival-regulatory role was later described in glioblastoma. In U87 glioblastoma cells, Rnd3 was shown to interfere with Rb inactivation and to modulate both proliferation and survival, linking its function to core cell-cycle and survival pathways beyond cytoskeletal regulation [30]. More recently, it was shown that in glioblastoma, reduced Rnd3 abundance is associated with impaired apoptotic competence, whereas its overexpression increases caspase-3 activation and cell death. Mechanistically, Rnd3 promotes ubiquitin-dependent proteasomal degradation of the NF-κB p65 subunit, thereby attenuating survival signaling and anti-apoptotic gene expression [10].

Epigenetic repression of RND3 also contributes to apoptotic control in glioma. HOXA-AS2 recruits EZH2 to the RND3 promoter and suppresses its transcription; accordingly, HOXA-AS2 depletion increases apoptosis and reduces proliferation and invasion, effects that are partially reversed by concomitant RND3 silencing. These findings support Rnd3 as a functional mediator of apoptotic competence downstream of the HOXA-AS2/EZH2 regulatory axis [17]. In esophageal squamous cell carcinoma, Rnd3 overexpression inhibited cell growth and promoted apoptosis, at least partly by attenuating EGFR signaling and ERK phosphorylation, suggesting that Rnd3 can favor apoptotic competence by limiting receptor tyrosine kinase-dependent survival pathways [26].

### 3.2. Rnd3 and Resistance to Anticancer Treatment

Beyond apoptosis regulation, several studies indicate that loss of Rnd3 contributes to therapy. One of the strongest examples comes from bladder cancer. Cao et al. identified a SIRT7/EZH2-dependent mechanism that represses RND3 and contributes to cisplatin resistance. SIRT7 reduces H3K18 acetylation and EZH2 increases H3K27 trimethylation at the RND3 promoter, leading to reduced RND3 expression. Functionally, RND3 knockdown increases cell viability, clonogenic capacity and cisplatin resistance, whereas RND3 overexpression enhances cisplatin sensitivity [16]. This study provides direct evidence that epigenetic silencing of RND3 can promote resistance to platinum-based therapy.

In hepatocellular carcinoma, reduced Rnd3 function promotes resistance to cisplatin and doxorubicin through ROCK2-dependent activation of the NF-κB/IL-6/STAT3 pathway. This loss of Rnd3 activity links therapeutic resistance to inflammatory survival signaling and invasive behavior, suggesting that impaired Rnd3 function may coordinate several aggressive tumor traits [29]. More recent work extends this concept to targeted therapy: sorafenib downregulates Rnd3 through Raf/MEK/ERK inhibition, thus relieving RhoA/ROCK activity and activating a FAK/AKT/HMGCR-dependent cholesterol biosynthesis program that promotes sorafenib resistance [14]. This finding extends the role of Rnd3 beyond classical apoptosis-related survival pathways, suggesting that loss of Rnd3 may also support therapy adaptation through metabolic rewiring.

Rnd3 has also been associated with platinum resistance in lung cancer. In cisplatin-resistant A549/DDP cells, inhibition of miR-196a increased Rnd3 protein abundance and was accompanied by reduced cisplatin resistance, decreased expression of drug-resistance-associated proteins and enhanced late-stage apoptosis [31]. Although these findings are limited to a cell-line model and do not establish a direct causal role for Rnd3, they suggest that higher Rnd3 protein levels may contribute to the response induced by miR-196a inhibition.

However, the relationship between Rnd3 and therapeutic response is not uniform across tumor types. In KATO III gastric cancer cells, combined miR-200c replacement and cisplatin treatment reduced RND3 mRNA and Rnd3 protein levels while increasing apoptosis, inhibiting migration and enhancing cisplatin sensitivity [32]. Because miR-200c simultaneously affected several genes involved in survival and invasion, and no RND3-specific rescue experiment was performed, these findings do not establish a direct causal role for Rnd3 in cisplatin resistance. Rather, they suggest that reduced Rnd3 levels may form part of a broader miR-200c-dependent sensitization program in this gastric cancer model. Taken together, the strongest functional evidence supports Rnd3 as a promoter of apoptotic competence and therapeutic sensitivity in glioblastoma, bladder cancer and hepatocellular carcinoma. Additional cell-line studies in lung and gastric cancer suggest that its contribution to drug response is more context-dependent and may be embedded within broader microRNA-regulated networks. Rnd3 should therefore not be considered a universal inducer of apoptosis or a uniform sensitizer to therapy. Reduced RND3 expression or impaired Rnd3 function frequently favors tumor cell survival and treatment resistance, but the final outcome depends on tumor lineage, therapeutic pressure and the dominant survival network (Table 1).

## 4. Rnd3 in Migration, Invasion and Epithelial–Mesenchymal Plasticity

**Figure 3:**
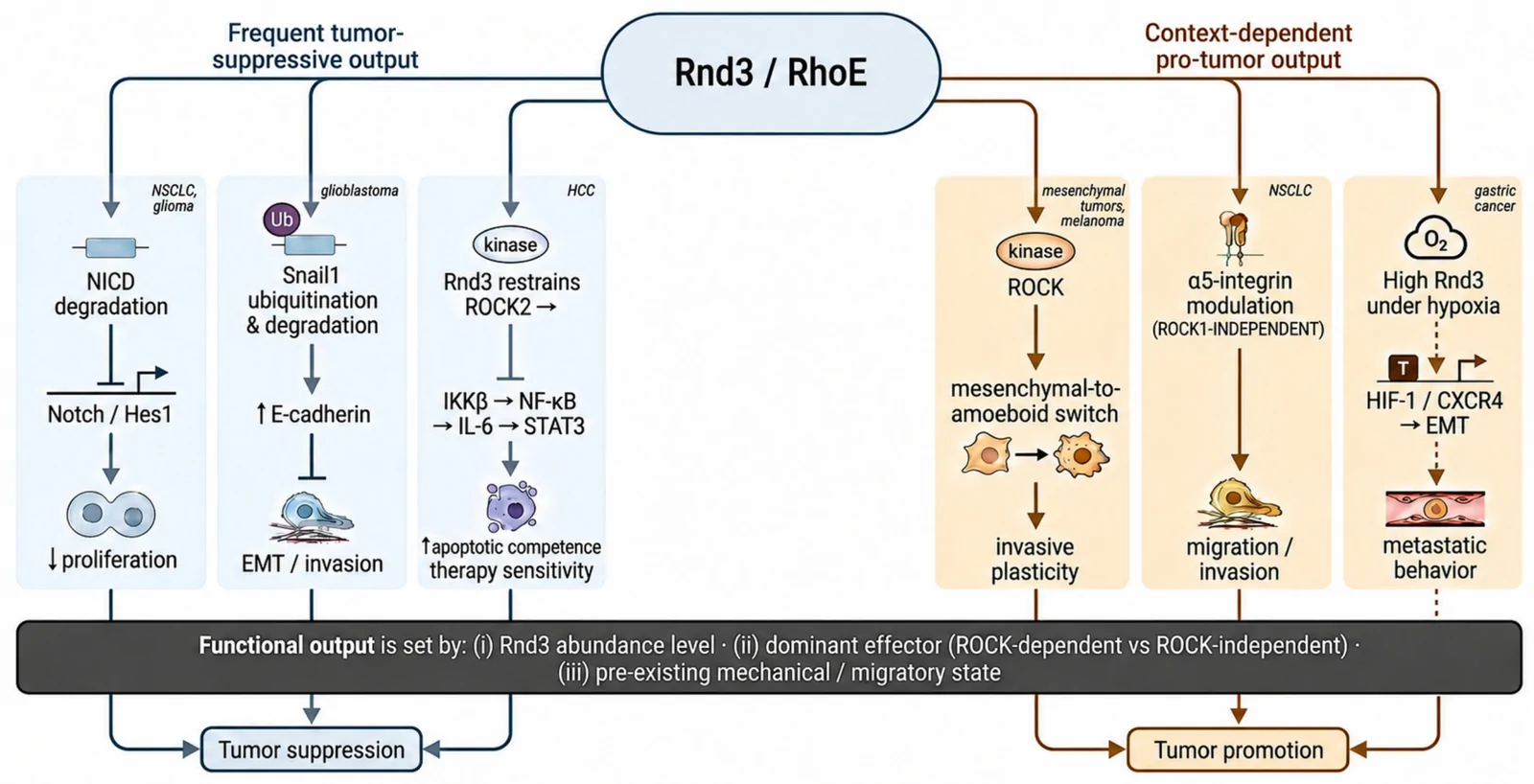
Molecular basis of the context-dependent duality of Rnd3. Depending on cellular context, the same atypical, abundance-regulated GTPase Rnd3/RhoE produces opposite functional outputs. Tumor-suppressive branches (**left**, blue) include Notch/Hes1 inhibition through NICD degradation, Snail1 ubiquitination and degradation with E-cadherin restoration and restraint of ROCK2–IKKβ–NF-κB–IL-6–STAT3 signaling that increases apoptotic competence and therapy sensitivity. Context-dependent pro-tumor branches (**right**, orange) include ROCK-dependent mesenchymal-to-amoeboid plasticity, ROCK1-independent α5-integrin-driven migration and invasion, and HIF-1/CXCR4-associated metastatic behavior under hypoxia. The net functional output is determined by Rnd3 abundance, the dominant downstream effector (ROCK-dependent vs. ROCK-independent) and the pre-existing mechanical state of the cell. Solid connectors denote functional (gain-/loss-of-function) evidence; the dashed connector denotes associative evidence. Rnd3 denotes the protein. Abbreviations: EMT, epithelial–mesenchymal transition; HCC, hepatocellular carcinoma; HIF-1, hypoxia-inducible factor 1; NICD, Notch intracellular domain; NSCLC, non-small-cell lung cancer.

The role of RND3/Rnd3 in migration, invasion and epithelial–mesenchymal plasticity is one of the most complex aspects of its cancer biology. In contrast to proliferation or apoptosis, where Rnd3 is more frequently reported as a tumor-suppressive regulator, invasion is not governed by a single cellular program. Tumor cells may migrate through mesenchymal, amoeboid or hybrid modes, and these modes depend on actomyosin contractility, focal adhesion dynamics and extracellular matrix interactions, among other determinants [12,34,35]. The studies discussed below assay Rnd3 at different levels—some scoring two-dimensional motility, others three-dimensional invasion, EMT markers or metastatic spread—so the direction reported for Rnd3 belongs to the level actually tested and does not automatically extend to the others. Because Rnd3 directly modulates cytoskeletal and mechanical signaling, its effects on invasion are intrinsically state-dependent [13,36]. Accordingly, Rnd3 can suppress invasive conversion in some contexts, while facilitating migration or infiltrative behavior in others [11,13,37]. These opposing invasive outputs can be integrated into a single mechanistic model, in which the same RhoA/ROCK-inhibitory activity of Rnd3 is directed toward suppression or promotion of invasion according to Rnd3 abundance, the dominant downstream effector and the pre-existing mechanical state of the tumor cell (Figure 3). This context dependence is evident across several tumor models in which Rnd3 has been linked to opposite invasive outputs. In hepatocellular carcinoma, reduced RND3 mRNA expression and lower Rnd3 protein levels were observed in tumor samples, particularly in invasive tumors with satellite nodules. Functionally, RND3 silencing increased three-dimensional motility and invasion and induced EMT-associated changes, including loss of E-cadherin, increased ZEB2 expression, and reduced miR-200b/c levels [38]. Consistent with these findings, reduced Rnd3 protein levels in HCC tissues were associated with tumor progression, higher tumor grade and poor patient outcome [39].

In gastric cancer, however, studies pointed in the opposite direction. Under hypoxic conditions, HIF-1-mediated upregulation of RND3 expression promotes epithelial–mesenchymal transition, indicating that Rnd3 may participate in hypoxia-driven EMT in gastric cancer cells [40]. Rnd3 was subsequently shown to promote migration, invasion and metastasis through a mechanism involving increased CXCR4 expression [41]. Together, these findings indicate that Rnd3 can exert pro-invasive effects when integrated into hypoxia- or CXCR4-driven metastatic programs.

Melanoma studies provided another major antecedent for the pro-migratory function of Rnd3. Klein and Aplin showed that Rnd3 regulates actin cytoskeleton and promotes melanoma migration and invasive outgrowth in three-dimensional environments [42]. Mechanistically, earlier work had already linked BRAF-dependent regulation of Rnd3 to actin cytoskeletal and focal adhesion organization [43]. Later, Klein and Higgins showed that a switch in Rnd3–RhoA signaling is critical for melanoma invasion after mutant BRAF inhibition, indicating that therapeutic rewiring can change the functional output of the RND3–RHOA axis [33,44]. Together, these melanoma studies are central to the interpretation that Rnd3 may facilitate invasion by preserving cytoskeletal plasticity in highly motile tumor states.

Additional studies reinforced the invasion-suppressive side of Rnd3 biology. In mesenchymal tumor cells, reduced RND3 expression was associated with increased invasiveness and metastatic potential, consistent with enhanced ROCK-dependent amoeboid-like invasion [45]. In a PTEN/PI3K/Akt-associated esophageal squamous cell carcinoma model, reduced Rnd3 was associated with a tumor-suppressive effect [46]. In breast and liver cancer cell models, AES-dependent maintenance of RND3 expression restrained proliferation and invasion, suggesting that loss of RND3 activity can contribute to malignant progression [47]. Finally, network-based and functional analysis in glioma implicated Rnd3 in multiple hallmarks of cancer, including migration and invasion, indicating that even within brain tumors Rnd3 cannot be interpreted through a single suppressive model [24].

Together, these studies show that Rnd3 can either suppress or promote invasive behavior depending on the cellular and signaling state, rather than on tumor lineage. A suppressive direction has been reported in HCC, mesenchymal and selected carcinoma models, whereas a pro-invasive direction has been reported under hypoxia in gastric cancer models and in BRAF-driven melanoma models. This duality reflects the role of Rnd3 in regulating the mechanical and signaling states that determine how tumor cells move.

### 4.1. Rnd3 as an Invasion Suppressor: Snail1, RhoA/ROCK Restriction and Loss of Epithelial Control

Several recent studies support an invasion-suppressive role for Rnd3. One of the clearest examples comes from glioblastoma where Rnd3 promotes Snail1 protein degradation and inhibits glioblastoma cell migration and invasion [11]. Because Snail1 is a canonical EMT-associated transcription factor, its Rnd3-dependent destabilization links Rnd3 activity to the preservation of epithelial features, adhesion control and reduced invasive behavior. By promoting Snail1 degradation, Rnd3 links cytoskeletal regulation to transcriptional control of invasive plasticity.

In glioma, this invasion-suppressive function is also supported by epigenetic regulation of RND3. As discussed above, Wu et al. (2019) showed that HOXA-AS2-mediated repression of RND3 contributes to malignant glioma behavior. In the same HOXA-AS2/EZH2 model, RND3 repression was also linked to invasion, since HOXA-AS2 knockdown reduced glioma cell invasion and this effect was partially reversed by RND3 interference [17]. These findings support the idea that epigenetic silencing of RND3 can also contribute to an invasive glioma phenotype.

Rnd3-mediated control of RhoA/ROCK signaling provides a second major invasion-suppressive mechanism. In rhabdomyosarcoma, the Rnd3/ROCK/ARHGAP25 pathway controls cell invasion through inhibition of Rac activity [12]. Low Rnd3 expression in aggressive rhabdomyosarcoma models permitted ROCK-dependent signaling changes that favored an invasive phenotype, indicating that Rnd3 regulates not only the extent of invasion but also the mode of migration. These findings are consistent with a model in which reduced Rnd3 permits ROCK-dependent invasive behavior with rounded-cell features.

Hepatocellular carcinoma provides additional evidence for an invasion-suppressive function of Rnd3. RND3 silencing enhances chemoresistance and aggressive behavior through ROCK2-dependent activation of the IKKβ/NF-κB/IL-6/STAT3 signaling axis [29]. More recently, KIAA1429-dependent m6A modification was shown to destabilize RND3 mRNA through YTHDC1, reducing Rnd3 protein levels and promoting HCC migration, invasion and metastasis [20]. Together with earlier evidence linking reduced RND3 expression and loss of Rnd3 function to EMT-associated invasion, these findings support a model in which RND3 repression facilitates HCC aggressiveness through altered RhoA/ROCK signaling, inflammatory survival pathways and post-transcriptional control of RND3 mRNA [20,29,38].

Viral and microRNA-mediated mechanisms also converge on RND3 repression as a pro-metastatic event. In Epstein–Barr-virus-associated nasopharyngeal carcinoma, EBV-miR-BART2-5p suppresses RND3, activates Rho signaling and enhances motility and metastasis [48]. Thus, EBV-associated nasopharyngeal carcinoma links viral microRNA-mediated RND3 repression with enhanced metastatic competence. Similarly, in environmental carcinogenesis models, miR-802-mediated repression of RND3 has been linked to lung cancer progression and metastatic behavior after PM2.5 exposure [49]. Together, these findings connect RND3-dependent motility with upstream viral and environmental regulatory inputs.

In breast cancer, HOXD9-induced UXT recruits EZH2 to the RND3 promoter and represses RND3 transcription, thereby reducing Rnd3 and promoting proliferation, migration and invasion. Functional rescue experiments indicated that restoration of Rnd3 through forced RND3 expression contributes to the antitumor effects observed after inhibition of the HOXD9–UXT–EZH2 axis [18]. These findings place RND3 downstream of an epigenetic regulatory circuit and support an invasion-suppressive function for Rnd3 in this tumor context.

Overall, the available evidence indicates that Rnd3 can suppress invasion through several convergent mechanisms that preserve cytoskeletal balance and epithelial control while limiting Rho-family signaling, thus limiting the acquisition of invasive and metastatic phenotypes.

### 4.2. Rnd3 as a Facilitator of Migration and Invasion in Specific Tumor States

Despite the evidence above, Rnd3 cannot be described as a universal invasion suppressor. In selected tumor states, Rnd3 is required to maintain the cytoskeletal flexibility or signaling configuration needed for migration and invasion.

A clear example of the context-dependent pro-invasive function of Rnd3 is observed in glioblastoma downstream of receptor tyrosine kinase signaling. RTK inhibition reduces Rnd3, activates RhoA/ROCK signaling, promotes stress-fiber formation and focal-adhesion maturation, and decreases cell motility and invasion. Consistently, RND3 silencing reproduces this rigid and poorly motile phenotype, supporting Rnd3 as a mediator of RTK-driven cytoskeletal plasticity and invasiveness [13]. In this setting, Rnd3 preserves a permissive mechanical state by limiting excessive contractility and adhesion stabilization. This function contrasts with its Snail1-dependent anti-invasive activity in other glioblastoma models, indicating that these apparently opposing effects reflect differences in the dominant invasive program rather than mutually exclusive functions.

Non-small-cell lung cancer also provides evidence for a pro-migratory and pro-invasive function of Rnd3. Tang and colleagues showed that miR-200b/c directly targets Rnd3 and that miR-200b/c expression or Rnd3 knockdown reduces proliferation and invasion in NSCLC cells, suggesting that Rnd3 can support malignant behavior in this context [19]. More recently, García-García and colleagues reported that Rnd3 regulates lung cancer cell migration and invasion independently of ROCK1 signaling through modulation of integrin alpha 5 [37]. These data indicate that Rnd3-dependent invasion in NSCLC is not reducible to the canonical RhoA/ROCK inhibitory axis and may involve integrin-mediated adhesion and migration programs.

Melanoma provides a particularly strong example of context-dependent pro-invasive Rnd3 activity. Previous work showed that Rnd3 supports melanoma migration and invasive outgrowth in three-dimensional environments and that mutant BRAF signaling can influence Rnd3-dependent cytoskeletal organization [42,43]. The subsequent observation that BRAF inhibition reshapes Rnd3–RhoA signaling and alters invasive behavior indicates that therapeutic pressure can shift the role of Rnd3 within the invasion machinery [44]. In melanoma, CD70 trimerization activates MAPK signaling, increases Rnd3 protein abundance and reduces ROCK1/MYPT1 phosphorylation, stress-fiber formation and focal-adhesion stability, changes that are associated with enhanced invasive behavior. Because RND3 was not directly manipulated, these findings implicate Rnd3 in the CD70–MAPK-associated invasive response without establishing it as the sole causal mediator [36]. In a separate melanoma model, WNT5A depletion altered Rnd3 protein levels and RhoA activity in a BRAF-dependent manner, promoting a rounded amoeboid phenotype in BRAF wild-type cells while producing the opposite response in BRAF^V600 cells [35]. Together, these studies indicate that changes in Rnd3 levels contribute to melanoma migratory plasticity, although their functional consequences depend on the upstream signaling and oncogenic context.

The pro-invasive role of Rnd3 is also supported by gastric cancer antecedents. Under hypoxia, HIF-1-mediated transcriptional upregulation of Rnd3 promotes EMT in gastric cancer cells [40], and Rnd3 promotes gastric cancer metastasis through CXCR4-dependent mechanisms [41]. These gastric cancer models place Rnd3 within microenvironmentally driven metastatic programs, particularly under hypoxic conditions.

### 4.3. Rnd3 and Epithelial–Mesenchymal or Amoeboid Plasticity

The apparently conflicting effects of Rnd3 on EMT and invasion are better understood if epithelial–mesenchymal plasticity is considered as a continuum rather than as a binary switch. Rnd3 does not simply promote or inhibit EMT. Instead, it modulates the cytoskeletal, adhesive and signaling thresholds that determine whether tumor cells preserve epithelial features, acquire mesenchymal traits, shift toward amoeboid movement or remain in hybrid invasive states.

This context dependence is evident in cervical cancer and HeLa cell models. Cheng et al. reported that miR-200b directly targets Rnd3 and that Rnd3 silencing inhibits EMT and migration, with increased epithelial features and reduced mesenchymal markers [50]. In contrast, Nishizuka et al. showed that Rnd3 is induced during TGF-β-mediated EMT and that Rnd3 knockdown enhances EMT-associated morphology, migration, Snail expression, fibronectin expression and RhoA activity; importantly, ROCK inhibition partially reversed these effects [51]. These findings appear contradictory only if Rnd3 is interpreted as intrinsically pro- or anti-EMT. A more coherent interpretation is that Rnd3 output depends on the signaling context in which EMT is engaged. Under TGF-β stimulation, Rnd3 may act as a compensatory brake on excessive RhoA/ROCK activation, whereas in other settings it may contribute to the cytoskeletal organization required for migration.

This context dependence also applies to the balance between mesenchymal and amoeboid invasion. Previous studies have linked reduced Rnd3 to ROCK-dependent invasive behavior and to changes in the mode of tumor cell migration [12,34]. Rather than regulating invasion as a simple on/off process, Rnd3 appears to influence how tumor cells invade by adjusting RhoA/ROCK activity, Rac-dependent signaling, focal adhesion dynamics and actomyosin contractility.

Overall, Rnd3 should be viewed as part of a mechanical plasticity module that connects EMT-associated transcriptional programs with cytoskeletal and adhesive states. Depending on the upstream signal and the pre-existing migratory phenotype, Rnd3 may either restrict invasive transition or preserve the mechanical flexibility required for tumor cell movement.

### 4.4. Clinical and Correlative Evidence Supporting Rnd3 Involvement in Invasive Programs

In addition to functional studies, clinical and transcriptomic analyses have associated Rnd3 with invasion, EMT-related programs and metastatic progression. In colorectal cancer, Rnd3 expression has been linked to relapse, depth of invasion, lymph node metastasis and distant metastasis [52]. More recent studies have connected miR-200 family dynamics and EMT-related target genes, including Rnd3, with tumor progression and metastatic transitions in this tumor type [53,54]. These observations suggest that Rnd3 expression may change across distinct stages or spatial contexts of colorectal cancer progression. However, they remain correlative and do not establish that Rnd3 directly drives EMT or metastasis.

In head and neck, laryngeal and hypopharyngeal tumors, studies associated Rnd3 with gene-expression programs related to motility, cytoskeletal remodeling and metastatic behavior [55,56,57].

Additional transcriptomic evidence supports a possible association between RND3 expression and treatment-related tumor cell states. In irradiated glioma cultures, combined treatment with radiation and the bi-(AID-1-T) aptamer increased RND3 mRNA expression while reducing proliferation and migration; however, neither Rnd3 protein level nor a direct functional contribution of RND3 was assessed [58]. Similarly, RND3 mRNA was strongly upregulated in TP53-null HCT116 colorectal cancer cells within a broader transcriptomic profile associated with potential multidrug-resistance mechanisms, although no direct role for Rnd3 was functionally validated [59]. In KATO III gastric cancer cells, combined miR-200c replacement and cisplatin treatment reduced RND3 mRNA expression together with migration and clonogenic growth, but the absence of RND3-specific rescue experiments and the multitarget activity of miR-200c preclude attributing these effects directly to Rnd3 [32].

Taken together, the evidence supports a dual but mechanistically coherent role of Rnd3 in migration, invasion and epithelial–mesenchymal plasticity. Rnd3 acts as an invasion suppressor when its main function is to destabilize Snail1, preserve epithelial or less invasive features, restrain RhoA/ROCK-driven contractility, limit ROCK-dependent rounded or amoeboid-like invasive behavior, or oppose prometastatic regulatory programs involving NF-κB/IL-6/STAT3 signaling or m6A-dependent repression of RND3. This pattern is supported by studies in models of glioblastoma, HCC, rhabdomyosarcoma, breast cancer, EBV-associated nasopharyngeal carcinoma, mesenchymal tumor cells and selected carcinomas.

Conversely, Rnd3 acts as a facilitator of invasion when tumor cells require reduced contractility, flexible actin organization, integrin-mediated adhesion, hypoxia-driven EMT, CXCR4 signaling, RTK-dependent motility, BRAF/MAPK-associated remodeling or melanoma-like invasive plasticity. This pattern is supported by studies in RTK-driven glioblastoma, NSCLC, BRAF-driven melanoma and hypoxic gastric cancer models. These determinants were not all assessed against the same endpoint, so the facilitator label groups observations drawn from motility, invasion and metastasis assays rather than a single, uniformly measured effect.

Therefore, Rnd3 should not be described as simply anti-EMT or pro-EMT. Its function is better conceptualized as that of a mechanical and signaling rheostat that tunes actomyosin contractility, focal adhesion dynamics, Rac/RhoA balance and EMT/amoeboid plasticity. Whether this tuning suppresses or promotes invasion depends on tumor lineage, oncogenic pathway status, microenvironmental cues, therapeutic pressure and the pre-existing migratory state of the tumor cell. For this rheostat model to become predictive, the pre-existing mechanical state it invokes must be defined through parameters that can be measured before or during Rnd3 perturbation, independently of the resulting phenotype. Several of these parameters are already present in the studies discussed above. RhoA/ROCK pathway activity can be monitored through ROCK-dependent phosphorylation of MYPT1 and MLC, together with actomyosin contractility and stress-fiber organization [7]. Focal-adhesion dynamics and maturation are reflected in the rigid, poorly motile phenotype produced by Rnd3 loss downstream of RTK signaling [13]. The Rac/RhoA balance is shifted by Rnd3 through the RhoE/ROCK/ARHGAP25 axis, which controls the mesenchymal or amoeboid mode of invasion [12], and cell-shape descriptors such as mitotic rounding and morphological elongation also depend on Rnd3 activity [60]. Extracellular matrix stiffness and cell-generated traction forces provide further quantifiable descriptors of the mechanical context, although their direct relationship with Rnd3 remains to be tested. None of these parameters defines the direction of Rnd3 function by itself. Each one characterizes, in a measurable and phenotype-independent way, the mechanical state in which the RhoA/ROCK-inhibitory activity of Rnd3 operates. Measuring this state before perturbation, rather than after observing the outcome, is what would give the model predictive value.

Across tumor types, the evidence indicates that lineage provides only a partial guide to Rnd3 function. Glioblastoma illustrates this particularly well, since Rnd3 can suppress Notch-dependent growth and Snail1-dependent invasion in some models while supporting RTK-driven motility in others. Similar context-dependent patterns are observed in HCC and NSCLC, where Rnd3 may either inhibit or support specific malignant programs depending on the dominant signaling and mechanical state. Thus, tumor type should be interpreted together with pathway activity, therapeutic pressure and cellular phenotype rather than used as a standalone predictor of Rnd3 function. This context-dependent view is further reinforced at the pan-cancer level. In a recent multi-omics characterization of Rho GTPases and their regulators across 33 cancer types, *RND3* was explicitly highlighted as a gene displaying opposite dysregulation directions depending on tumor context, frequently downregulated in hepatocellular carcinoma, consistent with a tumor-suppressive role, yet upregulated in gastric cancer, where it mediates drug resistance [61]. Such large-scale, cross-tumor evidence supports the notion that the functional output of Rnd3 cannot be inferred from tumor type alone but emerges from the dominant regulatory and signaling context.

## 5. Clinical Relevance and Biomarker Limitations

The clinical relevance of RND3/Rnd3 lies in its potential to inform tumor-state interpretation rather than in its use as an isolated biomarker. Across cancer types, changes in RND3 expression or Rnd3 levels have been associated with proliferation, invasion, metastatic behavior, apoptosis resistance and therapeutic response. However, the direction of these associations is not uniform: reduced RND3 expression may indicate loss of a suppressive constraint in some tumors, whereas preserved or increased Rnd3 activity may support motility, invasive adaptation or tumor cell fitness in others.

This context dependence limits the value of RND3 as a standalone prognostic or predictive marker. Its interpretation should therefore be integrated with tumor lineage, pathway activity, mechanical phenotype and treatment context. In this regard, RND3/Rnd3 may be most informative when evaluated together with pathway markers such as Notch, RhoA/ROCK, RTK/MAPK, EZH2/SIRT7, m6A-regulatory components, integrin-associated programs or EMT/plasticity markers. Future biomarker studies should distinguish correlative expression data from functional evidence and should prioritize patient-derived models, endogenous RND3 modulation and pathway-resolved validation.

## 6. Conclusions

This review revisits the apparent paradox of RND3/Rnd3 in cancer by moving beyond a binary oncogene versus tumor-suppressor interpretation. Its central contribution is to identify a mechanistic invariant, the conserved inhibition of RhoA/ROCK1-dependent contractility, that persists across contexts: the divergent phenotypes reported for Rnd3 reflect redirection of this conserved action by context-specific accessory effectors, not a reversal of the action itself. The available evidence indicates that Rnd3 acts as a context-dependent regulator of tumor cell state, linking cytoskeletal mechanics with proliferation, survival, therapeutic response and invasive plasticity. In many tumor models, Rnd3 restricts proliferation, promotes apoptotic competence or limits treatment resistance. However, in specific contexts, Rnd3 may support tumor fitness or invasion.

These apparently divergent findings can be organized into an evidence map (Table 2) that records, for each study, the pre-existing context, the type of perturbation and the strength of the causal evidence, rather than assigning a fixed role to Rnd3 in each tumor type. Read in this way, the same conserved action appears as a suppressive constraint in some settings and as support for tumor fitness or invasion in others, making contextual interpretation essential for future mechanistic and biomarker studies. Defining that context is an experimental task: it will require causal, context-resolved approaches that combine endogenous Rnd3 modulation with pathway readouts, three-dimensional invasion models, treatment-response assays and patient-derived systems to establish when Rnd3 restrains and when it supports tumor progression.

Where earlier overviews grouped these findings by tumor type, the present review holds the conserved molecular action fixed and treats context as what redirects it; from this standpoint, the contexts that remain untested are not a marginal caveat but a principal result. With Rnd3’s action on RhoA/ROCK1 already well established, what is left to determine is which measurable states redirect it, and the studies compiled here show how rarely those states have been recorded. Characterizing them—baseline RhoA/ROCK activity, dominant pathway, mechanical state of the matrix and prior therapeutic exposure—is the experimental task that would begin to resolve the functional paradox of Rnd3 into a question of the measurable states in which its conserved action operates.

## Figures and Tables

**Table 1:** Relationship between RND3/Rnd3 status and anticancer treatment response. Reported associations between RND3/Rnd3 status and response to anticancer therapies, grouped by therapy class. Most relationships are associative rather than a direct drug effect on Rnd3. Therapy classes with no retrieved study are shown as explicit evidence gaps.

Tumor Type	Therapy/Therapeutic Context	Effect on Rnd3	Downstream Mechanism	Effect on Therapeutic Response	Evidence Level	Reference
**Bladder cancer**	Cisplatin (chemotherapy)	Baseline SIRT7/EZH2-mediated RND3 repression associated with cisplatin resistance	SIRT7 lowers H3K18ac and EZH2 raises H3K27me3 at the *RND3* promoter	*RND3* loss promotes cisplatin resistance; overexpression restores sensitivity	Direct functional (gain- and loss-of-function)	Cao et al., 2024 [16]
**Hepatocellular carcinoma**	Cisplatin (chemotherapy)	Reduced Rnd3 activity associated with resistance	ROCK2-dependent activation of NF-κB/IL-6/STAT3	Increased chemoresistance	Direct functional	Ma et al., 2016 [29]
**Hepatocellular carcinoma**	Doxorubicin (chemotherapy)	Reduced Rnd3 activity associated with resistance	ROCK2-dependent activation of NF-κB/IL-6/STAT3	Increased chemoresistance	Direct functional	Ma et al., 2016 [29]
**Hepatocellular carcinoma**	Sorafenib (targeted therapy)	Sorafenib downregulates *Rnd3*	Raf/MEK/ERK inhibition → RhoA/ROCK activation → FAK/AKT/HMGCR-dependent cholesterol biosynthesis	Promotes sorafenib resistance	Direct mechanistic	Feng et al., 2025 [14]
**Melanoma (BRAF V600E)**	MAPK inhibition, BRAFi + MEKi/avutometinib (targeted therapy)	MAPK inhibition downregulates *RhoE/RND3*	*RhoE* loss activates RhoA-FAK-AKT (adaptive resistance); FAK inhibition reverses it	Promotes adaptive resistance; avutometinib + FAKi overcomes it	Direct functional (PDX + syngeneic)	Lubrano et al., 2025 [33]
**NSCLC (A549/DDP)**	Cisplatin resistance model (chemotherapy)	miR-196a inhibition increases *Rnd3* protein	Associated with lower resistance-related proteins and enhanced apoptosis	Reduced cisplatin resistance	Indirect (no *Rnd3*-specific rescue)	Li et al., 2016 [31]
**Gastric cancer (KATO III)**	Cisplatin + miR-200c replacement (chemotherapy)	Reduced *RND3* mRNA and *Rnd3* protein	Part of a broader miR-200c-regulated program	Increased cisplatin sensitivity and apoptosis	Associative only	Ghasabi et al., 2019 [32]
**Radiotherapy**	—	No evidence identified	No evidence identified	No evidence identified	—	—
**Immunotherapy**	—	No evidence identified	No evidence identified	No evidence identified	—	—
**Hormonal therapy**	—	No evidence identified	No evidence identified	No evidence identified	—	—

Nomenclature: *RND3* (gene/mRNA), *Rnd3* (protein). Evidence level distinguishes direct functional evidence (gain-/loss-of-function or rescue establishing an RND3/Rnd3-specific effect) from indirect or associative findings, in which RND3/Rnd3 changes accompany the response without RND3/Rnd3-specific causal demonstration. “No evidence identified” indicates that a literature search without date restriction retrieved no study in which the therapy class modulates RND3/Rnd3; these categories are reported as explicit evidence gaps.

**Table 2:** Evidence map of RND3/Rnd3 studies across human cancers. Each row summarizes an individual study according to tumor/model, direction of effect, pre-existing context, RND3/Rnd3 perturbation, experimental system, measured outcome, causal evidence, mechanism and main limitation. The direction of effect refers only to the specific model and endpoint analyzed in each study, not to an intrinsic role of Rnd3 in that tumor type. Direction-of-effect categories include suppressor, facilitator, dual and boundary; the qualifier “(associative)” marks studies in which RND3/Rnd3 changes accompany the phenotype without established RND3/Rnd3-specific causality. Gene symbol RND3 denotes transcript-level events; protein symbol Rnd3 denotes protein-level function.

Tumor/Model	Direction of Effect	Pre-Existing Context	RND3/Rnd3 Status or Perturbation	Experimental System	Outcome Measured	Causal Evidence/Rescue	Mechanism	Main Limitation
**Glioma** Wu et al., 2019 [17]	**Suppressor**	High HOXA-AS2/EZH2 activity (regulatory background)	RND3 transcriptionally repressed; de-repression by HOXA-AS2 KD	Cell lines + clinical + in vivo	Proliferation; invasion; apoptosis	Rescue: RND3 interference reverses effect	HOXA-AS2/EZH2 silencing of RND3	Indirect (lncRNA/EZH2 axis); Rnd3 role partly inferred
**Glioma/high-grade glioma/GBM** Clarke et al., 2015 [24]	**Facilitator**	High-grade glioma/grade IV network state	RND3/Rnd3 ↑ in grade IV glioma; RNAi LoF validation	Cell lines (2D/3D)	Proliferation; survival; migration/invasion; angiogenesis; outcome	Network inference + LoF; no rescue	Rho-GTPase network linked to glioma hallmarks	Network-inference study; context = glioma grade/network state
**Glioblastoma** (U87) Poch et al., 2007 [30]	**Suppressor**	*Not reported*	Rnd3 modulation	Cell lines	Proliferation; survival	Correlative/mechanistic	Rb pathway	Limited models; baseline context undefined
**Glioblastoma** Liu et al., 2015 [9]	**Suppressor**	*Not reported*	RND3 low; overexpression (GoF)	Cell lines + xenograft	Proliferation; xenograft growth	GoF; mechanism-supported	Notch transcriptional inhibition	Overexpression may exceed physiological levels
**Glioblastoma** Liu et al., 2016 [11]	**Suppressor**	*Not reported*	RND3 low; Rnd3 restoration/overexpression and knockdown	Cell lines + in vivo	Migration/invasion; Snail1–E-cadherin	GoF + LoF; Snail1 mechanism	Snail1 degradation → E-cadherin restoration	Baseline mechanical state not measured
**Glioblastoma** Sun et al., 2019 [10]	**Pro-apoptotic** (**suppressor**)	*Not reported*	Rnd3 restoration (GoF)	Cell lines + clinical + in vivo	Apoptosis	GoF; mechanism-supported	NF-κB p65 degradation	Direction from restoration; context undefined
**Glioblastoma** (**RTK-driven**) Almarán et al., 2022 [13]	**Facilitator**	RTK-pathway activation (operative background)	LoF (shRNA) + GoF (overexpression)	Primary cells + cell lines (2D/3D)	Migration; invasion	LoF + GoF; ROCK rescue (H-1152)	RTK → Rnd3 → RhoA/ROCK antagonism	Context-dependent; single lineage
**Hepatocellular carcinoma** Grise et al., 2012 [38]	**Suppressor**	*Not reported*	Rnd3 low; loss (LoF)	Cell lines + clinical	Invasion	LoF; mechanism-supported	Partial EMT; E-cadherin/ZEB2/miR-200; Rac1-dependent invasion	Baseline context undefined
**Hepatocellular carcinoma** Ma et al., 2016 [29]	**Suppressor**	*Not reported*	Rnd3 loss (LoF)	Cell lines + clinical + in vivo	Chemoresistance; invasion	LoF + rescue (RhoA/ROCK2 KD)	ROCK2 → IKKβ/NF-κB/IL-6/STAT3	Direction inferred from loss; context undefined
**Hepatocellular carcinoma** Shan et al., 2024 [20]	**Suppressor**	*Not reported*	RND3 mRNA destabilized (m6A)	Cell lines + clinical + in vivo	Migration; metastasis	LoF (m6A); mechanism-supported	KIAA1429/m6A/YTHDC1 decay	Effect via mRNA stability; context undefined
**Hepatocellular carcinoma** Feng, 2025 [14]	**Suppressor**	Sorafenib exposure (treatment context)	Rnd3 loss (sorafenib-induced)	Cell lines + organoids + in vivo	Sorafenib resistance	LoF; mechanism-supported	RhoA/ROCK → FAK/AKT/HMGCR	Treatment-specific; single drug
**Hepatocellular carcinoma** Basbous et al., 2022 [27]	**Boundary** (**reversible**)	*Not reported*	RND3 depletion (LoF)	Cell lines + 3D + in vivo	Cell-cycle state (senescence)	LoF + restoration (reversible)	hTERT-dependent reversible senescence	Reversible/non-lethal output; not classical suppression
**NSCLC** Tang et al., 2014 [8]	**Suppressor**	*Not reported*	RND3 low; restoration (GoF)	Cell lines:(H358/H520/A549)	Proliferation	GoF; mechanism-supported	NICD degradation → Notch/Hes1 (ROCK-indep.)	Overexpression model; context undefined
**NSCLC/A549-DDP cisplatin-resistant cells**—Li et al., 2016 [31]	**Suppressor** (**associative**)	Cisplatin-resistant A549/DDP state; miR-196a inhibition context	Rnd3 increased after miR-196a inhibition; RND3 not directly manipulated	Cisplatin-resistant A549/DDP cell line	Cisplatin response; viability; late apoptosis; resistance markers	Functional for miR-196a inhibition; associative for Rnd3; no RND3 rescue	miR-196a inhibition ↑ Rnd3, ↓ MDR1/MRP1/ERCC1/survivin/Bcl-2, ↑ late apoptosis	Single resistant cell line; multitarget miRNA effect; no direct Rnd3 causality
**NSCLC** Tang et al., 2018 [19]	**Facilitator** (**pro-malignant**)	*Not reported*	Rnd3 high in tumor; knockdown (LoF)	Cell lines + clinical (40 biopsies)	Proliferation; invasion	LoF; correlative clinical support	miR-200b/c–Rnd3/RhoE–ERK1/2	Opposite direction to Tang 2014; model-dependent
**NSCLC** García-García et al., 2026 [37]	**Facilitator**	*Not reported*	Rnd3 depletion (LoF)	Cell lines (A549/H460) + clinical	Migration; invasion	LoF; ROCK1-independent mechanism	Integrin α5 (ROCK1-independent)	Single pathway; baseline context undefined
**Gastric cancer** Zhou et al., 2016 [21]	**Suppressor** (**associative**)	*Not reported*	Rnd3 protein degraded (CMA)	Cell lines + clinical + in vivo	Proliferation	LoF (CMA) + CMA inhibition rescue	LAMP2A chaperone-mediated autophagy	Protein-level only; context undefined
**Gastric cancer** Zhou et al., 2011 [40]	**Facilitator** (**associative**)	Hypoxia (measured microenvironmental context)	RND3 transcriptionally up-regulated under hypoxia; Rnd3 implicated in EMT	Cell lines (hypoxia)	EMT	HIF-1-dependent induction; EMT-associated evidence	HIF-1/RND3 promoter/EMT	No metastasis endpoint; context-switch incompletely tested
**Gastric cancer** Feng et al., 2013 [41]	**Facilitator**	*Not reported*	Rnd3 overexpression and downregulation	Cell lines + clinical + in vivo	Migration; invasion; metastatic potential	GoF + LoF; CXCR4 partial mediation	Rnd3 promotes migration, invasion and metastatic potential partly through CXCR4 up-regulation	CXCR4-dependent mechanism is partial; context not prospectively defined
**Gastric cancer/KATO III** Ghasabi et al., 2019 [32]	**Facilitator** (**associative**)	Cisplatin exposure + miR-200c replacement; gastric cancer cell-line context	RND3 expression suppressed after miR-200c replacement; RND3 not directly manipulated	KATO III gastric cancer cells	Apoptosis; migration; clonogenic growth; cell-cycle arrest; cisplatin sensitivity	Functional for miR-200c/cisplatin; associative for RND3/Rnd3; no RND3-specific rescue	miR-200c + cisplatin ↓ Rnd3/VEGFR/MMP9; ↑ apoptosis, ↓ migration	Multitarget miRNA; no RND3 rescue; Rnd3 causality unproven
**Bladder cancer** Cao et al., 2024 [16]	**Suppressor**	*Not reported*	RND3 repressed; KD and overexpression	Cell lines + clinical + in vivo	Viability; cisplatin resistance	LoF + GoF (bidirectional)	SIRT7 (H3K18ac ↓) + EZH2 (H3K27me3 ↑) at RND3 promoter	Baseline context undefined
**Breast cancer** Hu et al., 2022 [18]	**Suppressor**	*Not reported*	RND3 repressed; restoration rescue	Cell lines + clinical + in vivo	Proliferation; migration; invasion	Rescue by RND3 restoration	HOXD9/UXT/EZH2 represses RND3	Indirect via repressor axis
**ESCC** Zhao et al., 2012 [46]	**Suppressor**	*Not reported*	Rnd3 overexpression; reduced Rnd3 in ESCC context	Cell lines + clinical	Proliferation; apoptosis	Correlative/mechanistic	PTEN/PI3K/Akt modulation	Baseline context undefined
**ESCC** Wang et al., 2016 [26]	**Suppressor**	*Not reported*	Rnd3 low; overexpression (GoF)	Cell lines	Growth; apoptosis	GoF; mechanism-supported	↓ EGFR signaling, ↓ ERK	Overexpression model
**Melanoma** Klein and Aplin, 2009 [42]	**Facilitator**	3D matrix environment (context)	Rnd3 expression associated with invasive behavior; RND3/Rnd3 depletion used for functional validation	Cell lines (3D)	Migration; invasive outgrowth	Mechanistic (cytoskeletal)	Suppression of RhoA/ROCK-mediated actin organization; 3D invasive outgrowth	Direction depends on migratory mode/system
**Melanoma** Pich et al., 2016 [36]	**Facilitator** (**associative**)	CD70 trimerization/MAPK activation	Rnd3 ↑ after CD70 trimerization; no direct RND3 manipulation	Cell lines	Migration/invasion	Functional for CD70; associative/mechanistic for Rnd3	CD70–MAPK	No RND3 silencing/rescue; Rnd3 role inferred downstream of CD70
**Melanoma** Jobe et al., 2021 [35]	**Dual/BRAF-dependent plasticity**	BRAF status; WNT5A signaling level	RND3 expression altered downstream of WNT5A depletion	Cell lines	Amoeboid/mesenchymal morphology; invasion mode	Mechanistic	WNT5A/RhoA	RND3 is part of the pathway, but not necessarily the sole causal effector
**Colorectal carcinoma** Luo et al., 2012 [25]	**Suppressor**	*Not reported*	RND3 targeted by miR-17	Cell lines	Proliferation	LoF (miR-17); mechanism-supported	miR-17 targeting of RND3	Single miRNA axis
**Colorectal carcinoma** Zhou et al., 2013 [52]	**Facilitator** (**associative**)	*Not reported*	Rnd3 high (clinical)	Clinical cohort	Invasion depth; metastasis; relapse	Correlative (clinical) only	Association with invasion/metastasis/relapse	Purely correlative; no manipulation
**Rhabdomyosarcoma** Thuault et al., 2016 [12]	**Suppressor**	*Not reported*	Low Rnd3	Cell lines + clinical	Invasion	LoF; mechanism-supported	Low Rnd3 permits ROCKII–ARHGAP25-dependent Rac inhibition and ARMS invasion	Baseline context undefined
**Mesenchymal tumor cells** Belgiovine et al., 2010 [45]	**Suppressor**	*Not reported*	Rnd3 reduced on transformation	Cell lines + in vivo	Invasion (amoeboid)	LoF; mechanism-supported	Rnd3 loss → ROCK-dependent amoeboid invasion; ↑ metastatic potential	Mode-of-migration dependent
**Cervical** (**HeLa**) Cheng et al., 2016 [50]	**Facilitator**	miR-200b regulatory context	RND3 targeted by miR-200b; RND3 silencing	Cell lines	EMT; migration	miR-200b modulation + RND3 silencing; direct targeting evidence	miR-200b–RND3 axis	Cell-line study; miR-200b has multiple targets; no in vivo or metastatic endpoint
**Cervical** (**HeLa**) Nishizuka et al., 2019 [51]	**Suppressor** (**compensatory brake**)	TGF-β-induced EMT	Rnd3 induced by TGF-β; RND3 knockdown	Cell lines	EMT morphology; migration; EMT markers	LoF; ROCK inhibition partially reverses the enhanced EMT phenotype	RhoA/ROCK-dependent restraint	HeLa/TGF-β model; EMT and migration endpoints only; no in vivo metastasis validation
**Nasopharyngeal** (**EBV**) Jiang et al., 2020 [48]	**Suppressor**	EBV infection (viral context)	RND3 virus-suppressed	Clinical + in vitro + in vivo	Motility; metastasis	LoF (viral); mechanism-supported	EBV-miR-BART2-5p; Rho activation	Effect virus-mediated
**Prostate cancer/DU-145** Bektic et al., 2005 [28]	**Suppressor/pro-apoptotic**	*Not reported*	RND3/Rnd3 underexpressed; forced Rnd3 overexpression	Prostate tissue + cell lines; DU-145 GoF model	Cell-cycle arrest; apoptosis	GoF; no RND3/Rnd3-specific rescue	Rnd3 overexpression ↓ CDC2/cyclin B1, induces G2/M arrest and caspase-3 cleavage	Early overexpression study; no bidirectional/rescue data; context undefined

Direction of effect: Suppressor = Rnd3 loss/repression promotes the measured process or Rnd3 restoration opposes it; Facilitator = Rnd3 presence/up-regulation supports the measured process; Dual/context-dependent = opposite outputs across conditions or models; Boundary = reversible, non-lethal state change, such as senescence; the qualifier “(associative)” indicates that RND3/Rnd3 changes are associated with the phenotype but direct RND3/Rnd3-specific causality is not established. ↑, increased; ↓, decreased (level or activity of the indicated molecule or process); GoF, gain-of-function; KD, knockdown; LoF, loss-of-function. Causal evidence indicates whether the effect was supported by gain-/loss-of-function, rescue experiments, pathway inhibition or correlation only. “Not reported” indicates that the pre-existing context was not experimentally defined in the original study. EBV, Epstein–Barr virus; EMT, epithelial–mesenchymal transition; ESCC, esophageal squamous cell carcinoma; NPC, nasopharyngeal carcinoma; NSCLC, non-small-cell lung cancer; RMS, rhabdomyosarcoma; RTK, receptor tyrosine kinase.

## Data Availability

No new data were created or analyzed in this study. Data sharing is not applicable to this article.

## Abbreviations

The following abbreviations are used in this manuscript:

3′UTR	3′ Untranslated Region
AES	Amino-Terminal Enhancer of Split
AKT	AKT Serine/Threonine Kinase (Protein Kinase B)
ARHGAP25	Rho GTPase-Activating Protein 25
BRAF	B-Raf Proto-Oncogene, Serine/Threonine Kinase
CD70	CD70 Antigen (TNF Superfamily Member 7)
CMA	Chaperone-Mediated Autophagy
CXCR4	C-X-C Chemokine Receptor Type 4
EBV	Epstein–Barr Virus
EGFR	Epidermal Growth Factor Receptor
EMT	Epithelial–Mesenchymal Transition
ERK	Extracellular Signal-Regulated Kinase
EZH2	Enhancer of Zeste Homolog 2
FAK	Focal Adhesion Kinase
GDP	Guanosine Diphosphate
GTP	Guanosine Triphosphate
H3K18ac	Histone H3 Lysine 18 Acetylation
H3K27me3	Histone H3 Lysine 27 Trimethylation
HCC	Hepatocellular Carcinoma
Hes1	Hes Family BHLH Transcription Factor 1
HIF-1	Hypoxia-Inducible Factor 1
HMGCR	3-Hydroxy-3-Methylglutaryl-Coenzyme A Reductase
HOXA-AS2	HOXA Cluster Antisense RNA 2
HOXD9	Homeobox D9
hTERT	Human Telomerase Reverse Transcriptase
IKKβ	Inhibitor of Nuclear Factor Kappa-B Kinase Subunit Beta
IL-6	Interleukin-6
KIAA1429/VIRMA	Vir-Like m6A Methyltransferase-Associated Protein (VIRMA)
LAMP2A	Lysosome-Associated Membrane Protein 2A
m6A	N6-Methyladenosine
MAPK	Mitogen-Activated Protein Kinase
MEK	Mitogen-Activated Protein Kinase Kinase
miR	MicroRNA
MYPT1	Myosin Phosphatase Target Subunit 1
NF-κB	Nuclear Factor Kappa-Light-Chain-Enhancer of Activated B Cells
NICD	Notch Intracellular Domain
NSCLC	Non-Small-Cell Lung Cancer
PI3K	Phosphoinositide 3-Kinase
PKCα	Protein Kinase C Alpha
PM2.5	Particulate Matter ≤2.5 Micrometers
PTEN	Phosphatase and Tensin Homolog
Rac	Ras-related C3 Botulinum Toxin Substrate
RAF	Rapidly Accelerated Fibrosarcoma Kinase
Rb	Retinoblastoma Protein
RhoA	Ras Homolog Family Member A
Rnd3/RhoE	Rho Family GTPase 3 (protein)
ROCK1/2	Rho-Associated Coiled-Coil-Containing Protein Kinase 1/2
RTK	Receptor Tyrosine Kinase
SIRT7	Sirtuin 7
Skp2	S-Phase Kinase-Associated Protein 2
Snail1	Snail Family Transcriptional Repressor 1
STAT3	Signal Transducer and Activator of Transcription 3
TGF-β	Transforming Growth Factor Beta
TP53	Tumor Protein 53
UXT	Ubiquitously Expressed Prefoldin-Like Chaperone
WNT5A	Wnt Family Member 5A
YTHDC1	YTH Domain-Containing Protein 1
ZEB2	Zinc Finger E-Box-Binding Homeobox 2
